# Patient reported outcomes in a large cohort of patients receiving osteopathic care in the United Kingdom

**DOI:** 10.1371/journal.pone.0249719

**Published:** 2021-04-16

**Authors:** Carol Fawkes, Dawn Carnes

**Affiliations:** 1 Institute of Population Health Sciences, Barts and the London School of Medicine and Dentistry, Queen Mary University of London, London, United Kingdom; 2 University College of Osteopathy, London, United Kingdom; Rowan University School of Osteopathic Medicine, UNITED STATES

## Abstract

**Objective:**

The use of Patient Reported Outcome Measures (PROMs) to evaluate care is being advocated increasingly in clinical settings. Electronic data capture is both resource and environmentally friendly and convenient. This purpose of this study was to test and implement a nationwide system to collect routine PROM data from osteopathic patients using a web and mobile app.

**Methods:**

A prospective study design was used to monitor outcomes of care for patients attending osteopathic clinics. Demographic and service data were collected, the primary outcomes were the Bournemouth Questionnaire and a Global Rating of Change score. Data concerning patients’ satisfaction and experience of care were collected also. Data were collected at baseline, one week, and six weeks post-treatment.

**Results:**

A total of 1721 patients completed the PROM app questionnaire. The majority (65.8%) of patients who used the PROM app were between 40 and 69 years old with 11% being 70 years and over. At baseline 39.8% of patients reported they’d had their symptoms for 13 weeks or more. Low back pain was the most common symptom (55.8%). Patients reported high scores for both satisfaction and experience of osteopathic care: 88.1% were very satisfied at six weeks post-baseline and 93.5% reported very good experience at six weeks post-baseline. Data from the Global Rating of Change scale indicated that at one week post-baseline 89.1% of patients reported some measure of improvement, and at six weeks this figure rose to 92.8%. The mean sum score for the Bournemouth Questionnaire went from 30.8 at baseline to 13.3 at six weeks post-baseline. This represented a significant and clinically meaningful positive change score of 56.8%.

**Conclusion:**

The app was well-completed and the data very encouraging. These data will help to form the basis for standards of care for patients attending osteopathic practices.

## Introduction

Ongoing quality assessment is an implicit part of good patient care, and this can be achieved in many ways. Historically quality assessment has been achieved through monitoring of scientific measures *e*.*g*. clinical tests; clinical audit; and ongoing data collection. More recent initiatives have focussed on the introduction of patient-reported outcome measures (PROMs) and patient-reported experience measures (PREMs) [1].

Commissioners of United Kingdom (UK) health services and private UK health insurers increasingly require evidence of effectiveness before considering the purchase of services for patients. Meeting the demands of an evidence-informed culture represents both challenges and opportunities for clinicians irrespective of the care setting. However, it is important to try to meet this demand and organisations which have previously focussed entirely on the use of clinical trial data are looking now at broadening the use of routinely collected data [2].

Although many Patient Reported Outcome Measures (PROMs) have been developed over the past 30 years, their introduction into day-to-day practice, and their use with technology to support such data collection has been relatively recent [3]. Paperless data collection is being advocated increasingly to benefit from technological developments, to increase wider access among the population, and it acknowledges the environmental cost of continued paper use [4]. The use of apps (short for application software) is becoming more common in healthcare. Apps are computer programmes designed to work on either a smartphone, tablet computer, or other mobile device [5].

Electronic data capture including PROMs is a growing market in healthcare and many companies offer services for data capture. Whilst technology is an important part of any data collection process, it is the data content itself, and the process involved in data collection that is key to project success. When any new area of development is planned it is important to involve all stakeholders in the process, this PROM app was intended for osteopathic care it was designed for patient completion but with clinician involvement, therefore both sets of user views were important. Evaluation of the measurement properties of different PROMs needed consideration also to ensure their suitability for the settings in which they are used [6].

Osteopaths in the UK are regulated by Statute [7]. Osteopaths in the UK are mainly comprised of clinicians who currently undergo training to BSc or MSc level [8] who practise osteopathy, however some osteopaths are medically-qualified doctors who undergo part-time training over a period of 18 months [9] who can practise as an osteopath and as a medical physician. Osteopaths in the UK treat a wide variety of patients presenting with a range of symptoms which include musculoskeletal and non-musculoskeletal symptoms [10–14]. The aim of this study was to establish an electronic PROMs data capture system, to create a national electronic dataset of outcomes using PROMS from osteopathic patients. This dataset is intended to provide information about the osteopathic profession to patients, clinicians, and service commissioners. The national profile of PROM findings also allows osteopaths to compare their performance to a national standard and reflect upon different areas of their practice, it also provides data for patients concerning the outcomes of care in different practices.

## Materials and methods

This section describes, in brief, the development of the content of the PROMs app, its pilot process, and then finishes with a description of the post-pilot data collection: these findings are the main focus of this study. This PROMs data collection study was conducted in the UK and involved patient participants from the UK. Ethics approval for the study was obtained from the research ethics committee at Queen Mary University of London (QMERC2014/18).

### PROM app development

The PROM app content was developed through two qualitative studies with patients (n = 22) [15] and clinicians: osteopaths (n = 32), chiropractors (n = 10), and physiotherapists (n = 4) [16]. The patients were positive about the process and wanted to provide feedback: clinicians were worried about the additional burden on patients but could see the value if the app was ‘user-friendly’. A clinimetric review of PROMS was conducted and as a result we chose three PROMs for testing in the PROM app: the Bournemouth Questionnaire (BQ) [17], the Roland Morris Disability Questionnaire (RMDQ) and a Visual Analogue Scale (VAS) [18–21]. The PROM app was developed by the National Council for Osteopathic Research as part of a PhD study undertaken by Carol Fawkes [22]. The app is available free of charge on Google Play (android version) and the App Store (iOS version).

### PROM app pilot testing

Data from the two qualitative studies and the systematic review of measurement properties informed the content of the PROM app. The app development was based on a ‘Software as a Service’ (SaaS) model. A staged process was followed including defining the app objective, designing the content, development of the app by Clinvivo.com, testing of the app and disseminating the app into clinical practice. The software release cycle for apps includes a series of stages from initial development to its eventual release:

- Pre-alpha: all activities performed during the software development but prior to testing;
- Alpha: the first phase of software testing using white box techniques [23];
- Beta: the phase generally begins when the software feature is complete, and generally incorporates usability testing to address speed and performance issues [24, 25];
- Open and closed beta: closed beta versions of software have restricted accessibility based on the decisions of the developers. Open beta versions, by contrast, are tested by a much wider, informal group who are invited to report bugs [26, 27];
- Full release of the ‘stable’ or final user version.

The initial version of the PROM app underwent α- and β-testing, and data security assessment. The pre-alpha and alpha testing stages were undertaken by Clinivivo.com. A closed beta version was created and tested by a convenience sample of patients from different age groups and technical capabilities who were asked to provide feedback on their experience. During the pilot data collection phase using the app patients were asked to provide any feedback on access, speed issues, and usability of the app. These were fed back to Clinvivo.com who addressed some minor issues prior to the release of the ‘stable’ or final user version of the software for this study.

The pilot testing of the app involved three separate data collection streams to assess the feasibility of using the app, its responsiveness, and test-retest reliability of the PROMs [28]. Volunteer osteopaths recruited patients on a consecutive basis. Patients with symptoms of low back pain of duration 0–6 weeks were included in the responsiveness arm of the study (R), patients with chronic, stable (unchanging) symptoms of low back pain of duration 13 weeks or more were included in the test-retest arm of the study (T), and patients with chronic changeable or subacute symptoms were included in the feasibility arm of the study (F). Patients accessed the data collection system by i) visiting the PROM app weblink to submit their details directly online, or ii) by downloading the app directly on to their mobile phone or tablet device. The patients received automated emails /”push notifications” and email/push notification reminders to their device via the app asking them to complete the follow up questionnaires at one and six weeks post-baseline.

Thirty osteopaths participated in the pilot study and they recruited 257 patients. The pilot process took place between September, 2014 and June, 2015.

Responsiveness data indicated that all of the PROMS were responsive, had good test-re-test reliability and were feasible [28]. The RMDQ was poorly completed and was removed from the final version of the app. The Bournemouth Questionnaire (BQ) includes a question about patients’ which duplicated the VAS information, therefore the VAS was removed from the final version of the app.

### Post-pilot data collection

The PROM selected for the final version of the app was the BQ. It is a composite measure comprised of seven questions. The questions ask patients about the effect of symptoms on: their life generally, activities of daily life, work (both inside and outside of the home), social activities, anxiety, depression, and how well patients can cope with their symptoms. Each question can be scored from 0–10; the scores from each of the individual domains are summed to produce a single score *i*.*e*. the sum score. This can produce a minimum score of 0 and a maximum score of 70: higher scores indicate a greater negative effect of symptoms for patients. The BQ version we used has a single modification to the original version and has been used successfully in a clinical setting [29].

In addition to the PROM data, demographic data (age, sex and work status), access to appointments, the duration of symptoms, the primary reason for seeking treatment, and the site of all symptoms for this episode of care were collected at baseline. At follow-up, participants were asked to complete questions concerning patient experience, patient satisfaction, and a Global Rating of Change question [30]. Additional questions include how patients are funding their care, the number of treatments they had received between baseline and follow up appointments, and the Friends and Family Test [31]. The Friends and Family Test was launched in the National Health Service (NHS) in the UK in 2013 to see if patients were happy with their care, and is used as a measure of experience by the NHS and many UK health insurers.

Osteopaths in the UK were invited to participate in using the PROM app for the post-pilot study through articles in the osteopathic media, social media posts, and conference and local osteopathic group presentations. Data collection took place between September, 2015 and August, 2020 when volunteer osteopaths invited consecutive adult patients who were either new patients or former patients consulting with a new symptom episode. All eligible patients attending participating practices were invited to join the study. The only exclusion criteria for this study were that patients were under 18 years of age, or were unable to read and understand the patient information sheet provided prior to submitting any data. Patients were given an information sheet about the study including its purpose, the process of data collection (via weblink or smartphone), how their data were stored, and reassurance that their data would not be shared. The information sheet contained a unique identifier (UID) provided by their osteopath to log into the system. The UID contained the recruiting osteopath’s UID (the osteopath’s registration number with the regulator, the General Osteopathic Council) and the patient’s UID. Patients are asked to consent to joining the study before completing the questionnaire. The osteopaths had no direct access to any individual patient data, the study team were unaware of the patient’s identity, and all data were pseudo-anonymised. However, summary PROMs reports for the osteopaths from their combined patient data were made available after a minimum of 25 completed datasets had been received from patients. Patients’ data were recorded by the Information Technology (IT) company who developed the app (Clinvivo Ltd). The patients were aware that their data were anonymised to avoid social desirability response bias [32].

### Data management

Data were transferred from the app design company (Clinvivo) using the Open PGP protocol, and data were decrypted using Gpg4win software and Kleopatra (Intevation GmbH, Osnabrück, Germany) which is a certificate manager and a universal crypto graphical user interface. These data were transferred to an Excel spreadsheet to allow data analysis in Excel, and Statistical Package for the Social Sciences (SPSS; IBM, Washington) version 22.

### Data analysis

Data analysis included descriptive statistics (including means and frequencies), and an analysis of change scores relating to the PROM.

## Results

To date (August, 2020), a total of 3022 submissions (baseline, one week, and six week data) have been contributed by 1721 patients using the PROM app. A total of 425 osteopaths volunteered to collect PROM data in their practices. The response rate for all outcomes at one week was 44.9% (n = 773) and at six weeks was 30.7% (n = 528).

### Demographics

The majority (65.8%) of patients who used the PROM app were between 40 and 69 years old, with 31% being over 60 years old; they were predominantly female (61.1%) and employed (68.2%) or retired (21.2%) (Table 1).

**Table 1:** Profile of patients who have contributed PROMs data at baseline.

Demographic item	Percentage (N = 1721)
**Age**	
18–29	7.3% (n = 125)
30–39	15.9% (n = 274)
40–49	20.7% (n = 356)
50–59	24.9% (n = 429)
60–69	20.2% (n = 348)
70–79	9.7% (n = 167)
80–89	1.1% (n = 19)
90 years and over	0.2% (n = 3)
Prefer not to answer	0% (n = 0)
**Sex**	
Male	38.9% (n = 669)
Female	61.1% (n = 1052)
Prefer not to answer	0% (n = 0)
**Work status**	
Employed	68.2% (n = 1174)
Retired	21.2% (n = 365)
Long term sick	0.8% (n = 14)
Looking after home and family	3.0% (n = 52)
Full time education	3.0% (n = 52)
Unemployed	1.2% (n = 21)
Other	2.3% (n = 38)
Prefer not to answer	03.% (n = 5)

**Fig 1:**
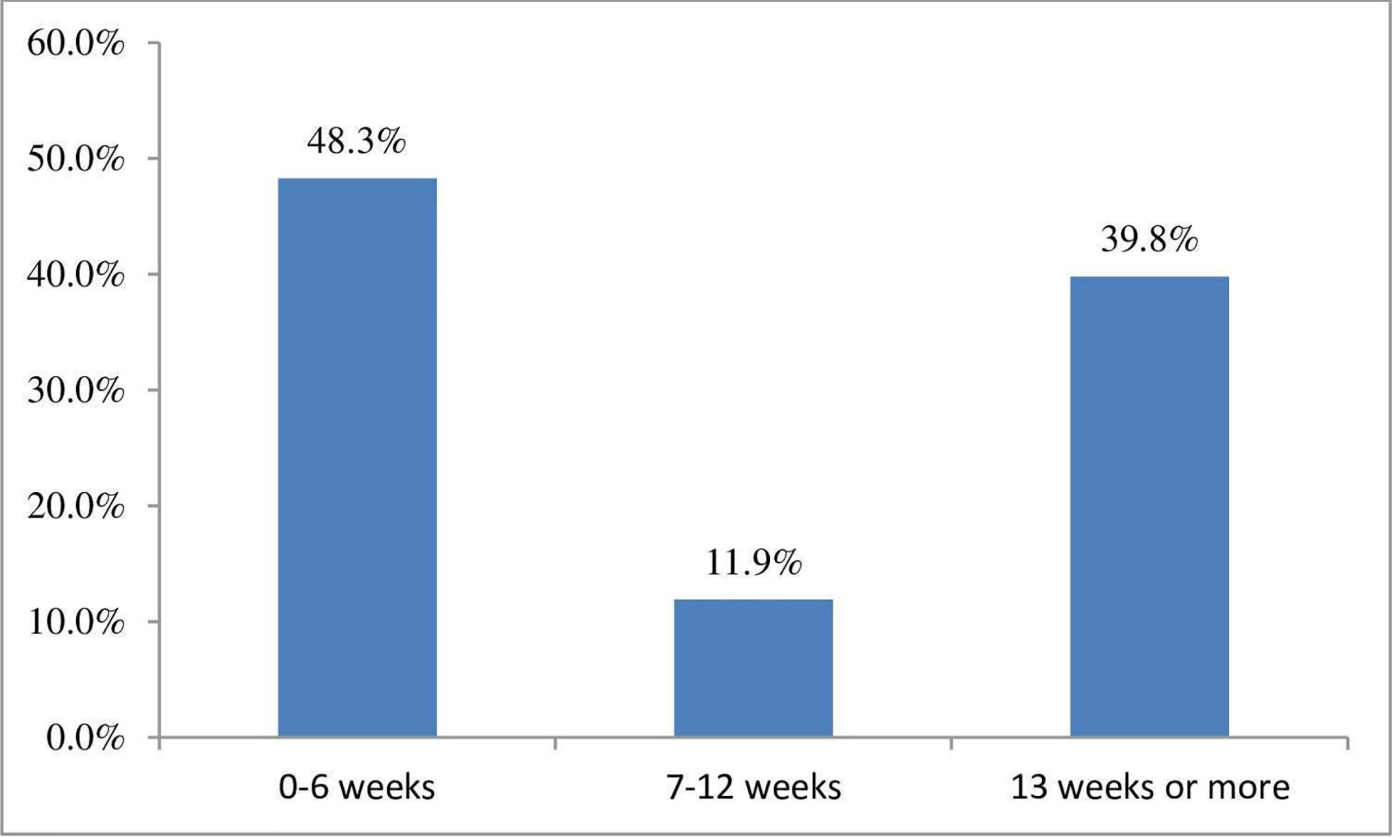
Duration of current symptoms for this symptom episode for patients presenting at their first appointment.

Patients reported waiting times for the first appointment to be offered to them. A total of 48.3% reported they were seen within 24 hours of contacting the practice, and 9.4% of patients reported waiting 7 days or more. Patients attended practices having experienced symptoms for different lengths of time. Acute presenting complaints (lasting 0–6 weeks) represented 48.3% of patients, and patients with chronic symptoms for this episode represented 39.8% (Fig 1).

**Fig 2:**
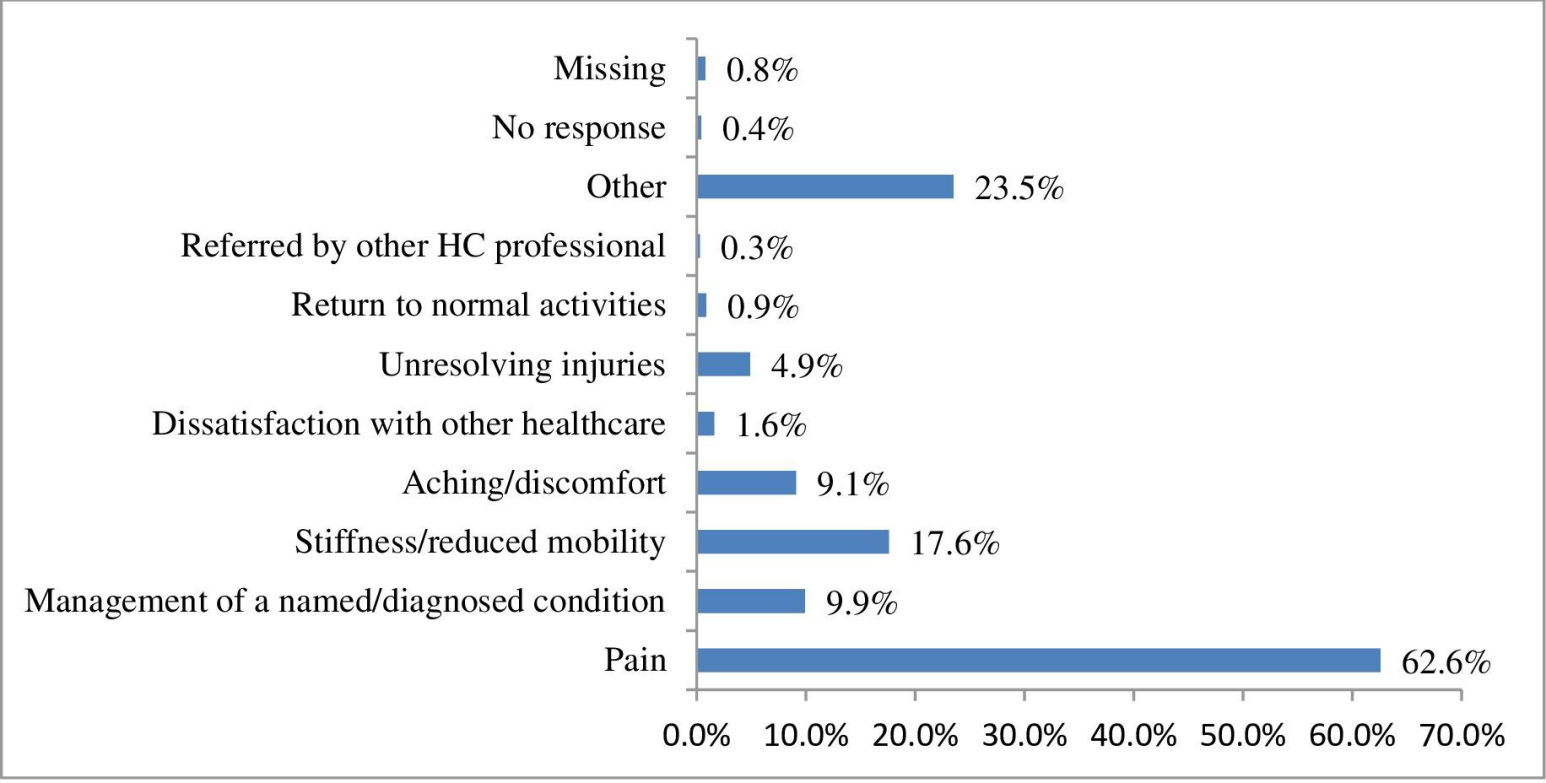
Reasons for seeking treatment.

Despite the variation in duration of symptoms, 89.2% of patients reported their general health as being either ‘good’ or ‘very good’. In contrast, 1.2% of patients reported their general health as being either ‘bad’ or ‘very bad’. The reasons for seeking treatment reported by patients varied (Fig 2). The most frequent reason was due to pain (62.6%), stiffness and mobility (17.6%), and other (23.5%), (this was a multi-response question so various reasons could be recorded).

**Fig 3:**
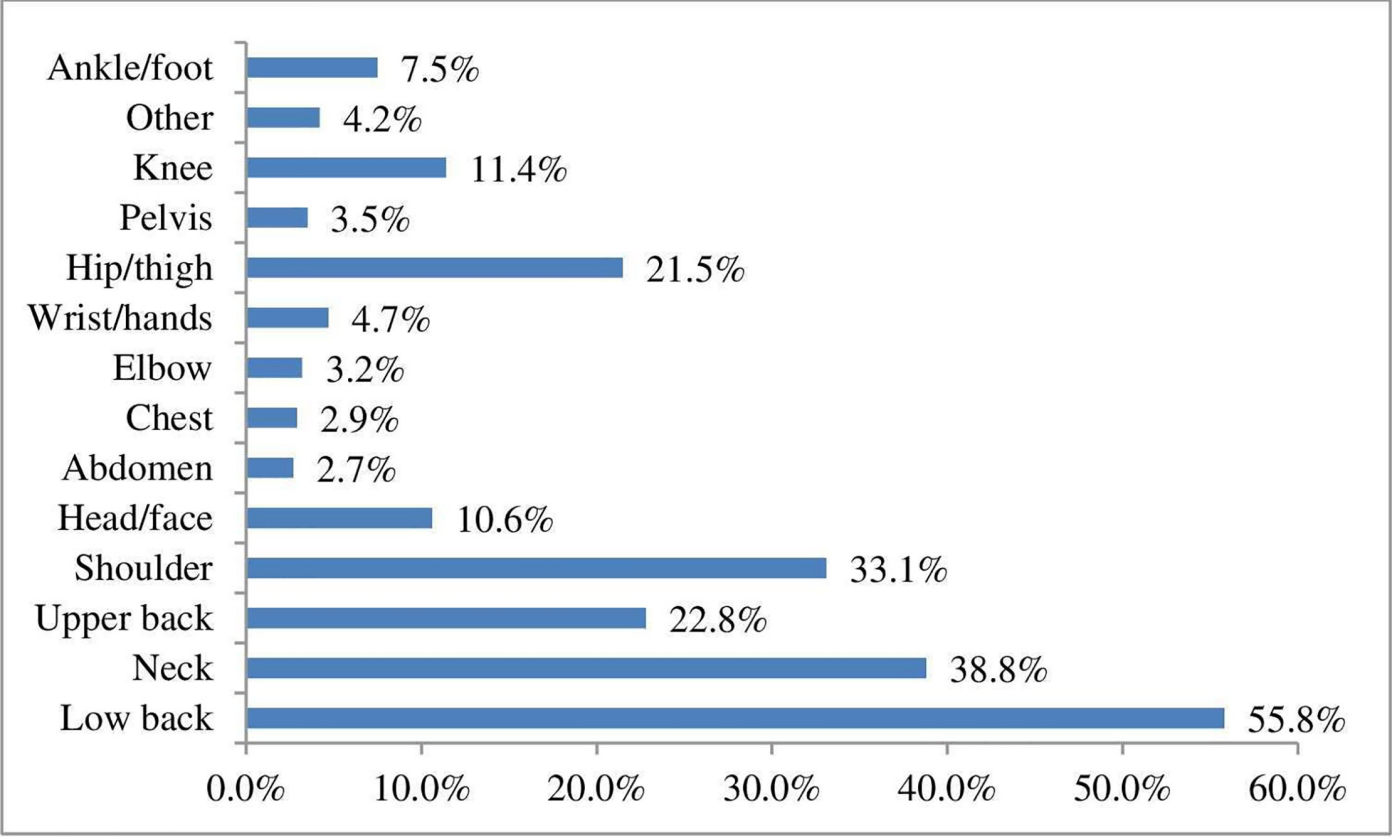
Symptom areas reported by patients at baseline.

Patients reported diverse areas for their symptoms (Fig 3). The most frequent area of symptoms was the low back (55.8%), followed by neck symptoms (38.8%), the shoulder (33.1%), and the hip/thigh (21.5%). Once again this was a multi-response question.

Patients reported their care was funded by themselves in 86.5% of cases. Additional sources of funding included health insurance (6.4%), the patient’s employer (0.9%), and a combination of different sources (2.3%). There was no response to this question from 3.9% of patients.

### Outcomes

Outcomes of care included satisfaction with osteopathic care, experience of care, and global change in addition to the change in score using the BQ.

#### Satisfaction

**Fig 4:**
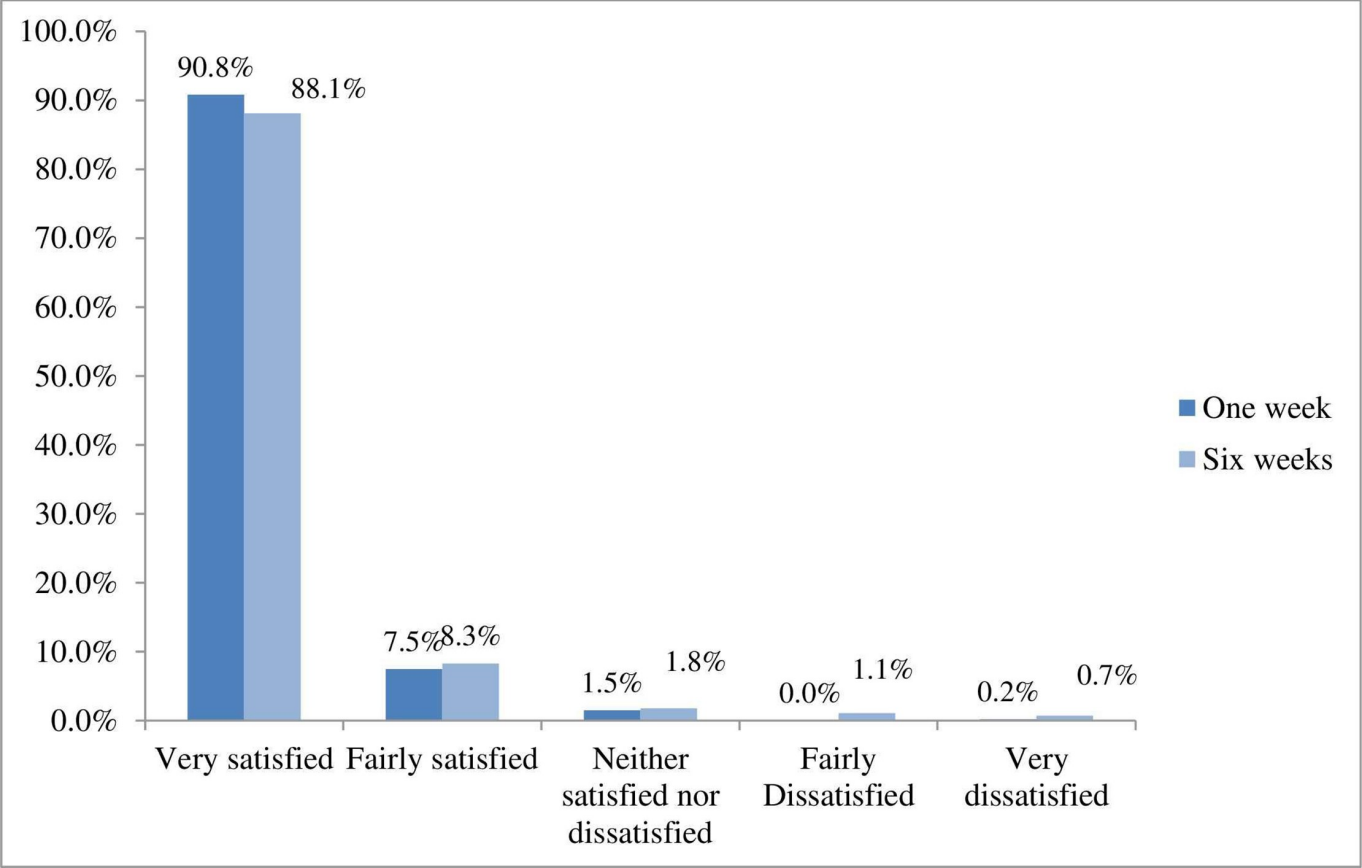
Patient satisfaction one week and six weeks post-treatment.

Patients reported they were ‘very satisfied’ with their care (90.8%) and ‘fairly satisfied’ (7.5%) at one week post-treatment. A small number reported being ‘fairly’ and ‘very dissatisfied’ at one week (0.2%) and six weeks (1.8%) as shown in Fig 4.

#### Experience

**Fig 5:**
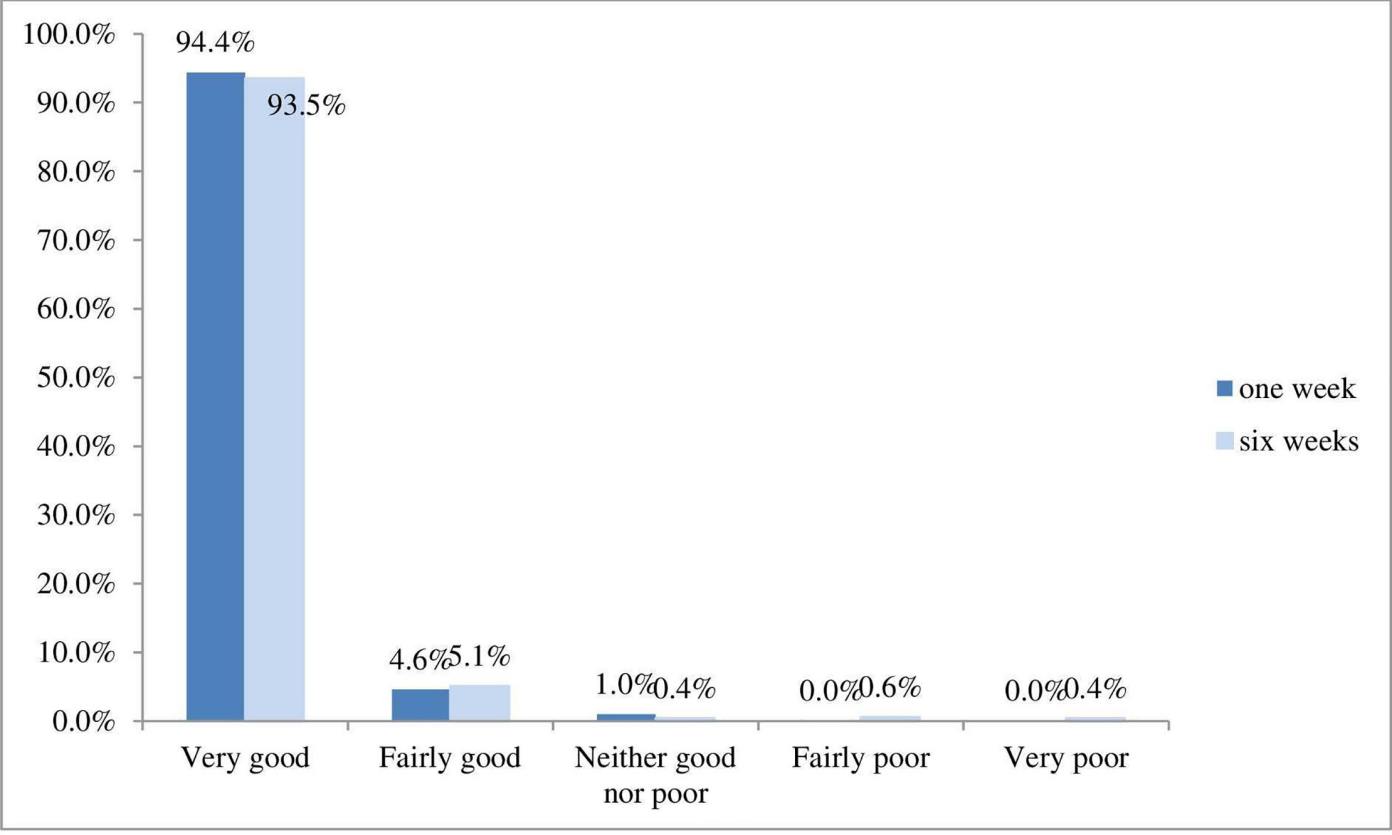
Patients’ experience of osteopathic care at one week and six weeks.

Patients’ experience of osteopathic care was generally good. Fig 5 shows that at one week (94.4%) and six weeks (93.5%) patients reported their experience of care was ‘very good’. At six weeks a small number (1%) reported their experience as being either ‘poor’ or ‘very poor’.

From mid 2019 we included the “Friends and Family Test” (n = 83) which indicated that 93.9% were ‘extremely likely’ or ‘likely’ to recommend osteopathy at one week and 78.2% at six weeks. A small number (4.4%) noted at six weeks they were ‘very unlikely’ to recommend osteopathic services

#### Global rating of change

**Fig 6:**
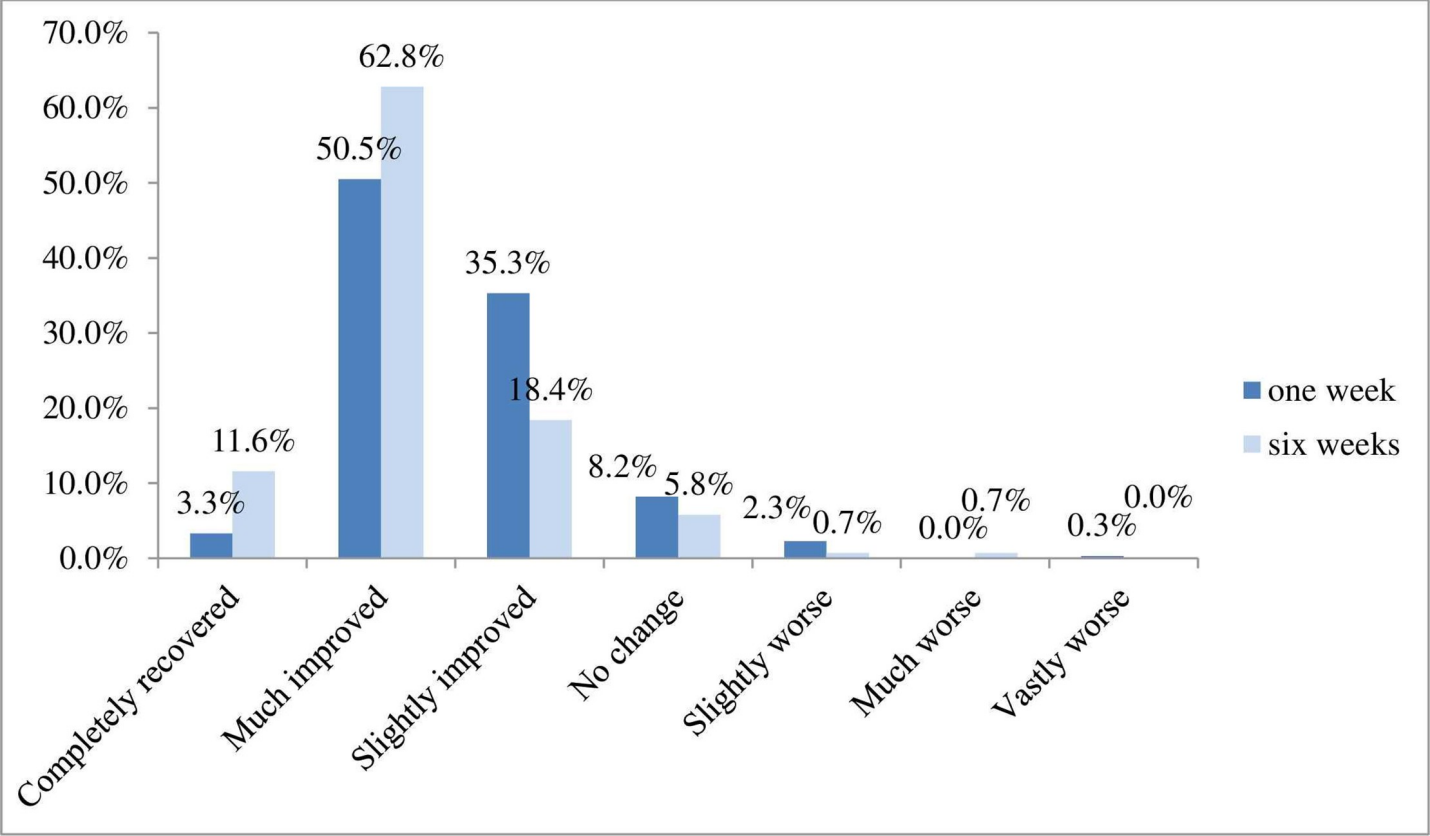
Global rating of change at one week and six weeks post treatment.

The change in patients’ symptoms indicated that many patients (89.1%) reported some measure of improvement at one week post-baseline, at six weeks this figure rose to 92.8% with 74.4% reporting they were ‘much improved’ or ‘completely recovered’ at 6 weeks post-baseline. A total of 8.2% reported ‘no change’ at one week and 5.8% at six weeks with 2.6% of patients reporting worsening at one week and 1.4% at 6 weeks (Fig 6).

#### Bournemouth questionnaire

Data submitted using the Bournemouth Questionnaire was generally well-completed (Table 2).

**Table 2:** Completeness of BQ data at all data collection points.

	Baseline	One week post-baseline	Six weeks post-baseline
Missing	n = 20	n = 3	n = 2
Incomplete	n = 35	n = 2	n = 1
Usable datasets	n = 1666	n = 768	n = 525

At baseline, the questions which were most frequently missed out were depression (n = 13), work (n = 10), anxiety (n = 8), and social activities and ability to control symptoms (2 each). At one week missed items included work (n = 2) and depression (n = 1), and at six weeks included work (n = 1) and depression (n = 1). Analysis of the BQ data (Table 3) showed a mean change score of 56.8% from baseline indicating significant meaningful change (Gurden *et al*., 2012).

**Table 3:** BQ data for each data collection point.

	Baseline (n = 1666)	One week post-treatment (n = 768)	Six weeks post-treatment (n = 525)
Mean sum score	30.8	17.0	13.3
Range	0–69	0–49.7	0–59.3

The percentage change score for the post-pilot data is:

$$\begin{array}{c} \mathrm{Percentage}\;\mathrm{change}=[(Baseline-discharge\;scores)÷Baseline]\times 100=\\ (30.8-13.3)÷30.8\\ =56.8\%. \end{array}$$

In their 2012 paper, Gurden *et al*. use an interpretation of the percentage change score:

■ ≤ 0% Deterioration of symptoms
■ 1–10% Small improvement
■ 11–30% Moderate improvement
■ > 30% Significant improvement

Data collection using the PROM app has yielded a considerable amount of useful data. There are areas for future development in the process.

## Discussion

### Summary of key points

The age distribution of respondents indicated that age was not a barrier to using the PROMs app either online (web app) or using a mobile device. The adult sample was skewed towards females and those who were in full time employment (either employed or self-employed). The presenting complaints were as expected for the profession (low back pain predominated) and patients were seen quickly; in nearly half the sample (48.3%) this occurred within 24 hours of contacting a practice. At six weeks post-baseline, patients they were ‘very’ or ‘fairly satisfied’ (96.4% ‘very satisfied’) and had a ‘good’ or ‘very good’ (93.5%) experience of osteopathic care, with 93.9% saying at one week they would be ‘likely’ or ‘extremely likely’ to recommend osteopathic care. This is the largest PROM dataset to provide information about global change for osteopathic care. Data showed that 89.1% of patients reported improvement at one week post-baseline, and 92.8% at six weeks post-baseline. Patients reported significant meaningful positive change (56.8%) from analysis of the BQ score.

Although only a small number of studies exist which profile osteopathic care, they do provide descriptive data to which this study can be compared. The majority of studies have included retrospective data collection [10–13] and one undertook prospective data collection [14]. The sample of patients in this study are very similar to previous studies on UK osteopathic populations and can be said to be representative of that population: the age ranges of respondents in previous studies were similar with the largest group of patients being in age range 41–60 (44%) in Pringle and Tyreman, 1993; 47% in the 35–54 age range in Burton, 1981; 40–49 in McIlwraith, 2003, and 22% in 30–39 in Fawkes *et al*., 2014. The gender distribution in this study was: 61.1% females and 38.9.1% males compared with 48% of females in Pringle and Tyreman; 49.8% of females in Burton; 37% females in McIlwraith; 60.5% in Hinkley and Drysdale, and 57% in Fawkes *et al*. [10–14]. All these previous studies used paper data collection methods, thus indicating little difference in age and gender profiles of respondents and predilection assumptions regarding the use of electronic data capture.

Clinical information about patients was similar to other studies in this field. Patients reported symptoms duration of 0–6 weeks in 48.3% of cases, 7–12 weeks in 11.9% of patients, and 13 weeks or more in 39.8%, indicating a mix of acute and chronic patients consistent with other studies [11, 13, 24]. Low back pain was the most common complaint among our responding patients (55,8%) this compares to 36% (Fawkes *et al*., 2014), 52% (Burton, 1981), 68% (McIlwraith, 2003), and 49% (Hinkley and Drysdale, 1995) [10–14].

The outcome data were encouraging. Our satisfaction data are comparable with other studies which have investigated patient satisfaction with osteopathic care which have also shown patient satisfaction to be high [33–37] even when outcomes of care have been smaller than anticipated [38]. The reasons for these findings have been explored and patients have noted good communication, clinician empathy, the opportunity to ask questions about their symptoms and their management, and competence. In a study of patient experience, (Drysdale *et al*.) 88.7% of patients reported good experience of osteopathic care either within osteopathic educational institutions or in private practices [39].

### Strengths and limitations

The use of the PROM app to collect independent outcome data was new to the osteopathic profession. It has the advantage of being patient rather than clinician completed. Earlier work in osteopathic care which collected outcome data relied upon completion of a VAS by the clinician, or in some cases by the patient in the presence of the clinician which limited the robustness of the data [14]. This study tried to reduce socially desirable responses but the distribution of data was positively skewed, either indicating very supportive data for osteopathic practice or that highly motivated satisfied patients were responsive or there was some selection bias in the sampling of patients by the osteopaths [32]. The design of the data collection study does not allow us to draw any conclusions regarding improvements which patients might have made as part of the natural history of their low back pain, or regression to the mean which must be considered when reviewing patients’ recovery [10, 40, 41]. However, it should be noted that there are various reasons for the use of PROMs in clinical practice and the findings on patients’ responses to treatment will help to identify new research questions for the future [42].

Recruitment of participants was generally slow throughout the study and this has impacted on the amount of data collected. Current use occurs in approximately 9% of the osteopathic profession. However, data collected have been from patients throughout the UK so it is representative of national practice. The routine collection of PROM data is one activity now advocated by the General Osteopathic Council, regulator for UK osteopaths [43]. This initiative may help increase engagement and offer patients the chance to provide their feedback about osteopathic care.

Response rates have been sub-optimal at 44.9% for one week and 30.7% at six weeks. Obtaining follow up data from participants is a challenging problem to address since patients cannot be contacted by a researcher to remind them about follow up data, and there is consideration of coercion if the osteopath also tries to remind patients about the follow up. The issues relating to follow up data using PROMs are widely recognised within healthcare but remain challenging to address in all settings [44].

Notwithstanding some of the challenges associated with PROM data collection, the post-pilot study has collected a good amount of data concerning the successful management of musculoskeletal symptoms. Musculoskeletal symptoms are very common both nationally and internationally representing a significant burden to the individual and healthcare funders [45, 46]. Since osteopathic techniques have been recognised in national guidelines, these data represent an opportunity for osteopaths to become more involved in managing the care of patients with musculoskeletal symptoms [47].

### Future directions

Data collection will continue using the PROM app but further developments will occur in time. Such development could centre on the inclusion of additional PROMs specific to different body sites, and for different conditions based on the findings from current PROM data. Changing the main PROM, the BQ, could also be considered in the future. At the time the PROM app was being developed, the Musculoskeletal Health Questionnaire was still evolving and was not an option for consideration [48]. As its use grows throughout musculoskeletal practice this would allow data from osteopaths to be comparted to other musculoskeletal services in the NHS provided by other Allied Health Professionals [49].

As the app development continues, the issue of whether data should be provided to osteopaths to review in real time to allow the patient responses to be used to modify care planning could be considered. Holmes *et al*. undertook a systematic review examining the effect of using PROMs in the management of patients with non-malignant pain [50]. They cited the poor quality, lack of generalisability and heterogeneity of the studies identified and concluded it was not possible to provide a comprehensive understanding of how PROMs may impact clinical treatment in this patient group. In a 2019 narrative review, Field *et al*. suggest that the routine use of PROMs during a course of care and not just at the baseline and end of treatment provides additional opportunity to inform clinician and patient with benefits for both [51].

To date, the app content has been translated into French, German, Spanish, Greek, Swedish, Dutch, and Danish. Translation into Norwegian is ongoing. Pilot data collection is occurring in these countries to complete the cross-cultural validation of the BQ [52].

Osteopaths in the UK treat patients of all ages. The PROM app was piloted in a separate study for data collection in a paediatric population. The original version of the app was not suitable for this purpose (based on feedback from parents and clinicians) therefore revisions were made to the app after consultation with different stakeholders and a second pilot was performed on the revised paediatric version of the PROM. Paediatric data collection is ongoing and findings concerning paediatric practice will be published when sufficient data have been submitted.

## Conclusions

The app performed well during the pilot phase from a functional perspective giving confidence in its use in the post-pilot study. Analysis of the post-pilot data identified that questions were answered well and with good completeness suggesting the content is not regarded as burdensome by patients. The patient reported outcome data were very positive. The main focus now is to increase engagement with osteopaths in private practice and to implement the app more widely. It is the right of every patient to be able to give feedback about their treatment and let their voice be heard. The PROM app is one way to achieve this in osteopathy.

## Supporting information

S1 Data (XLSX)

S2 Data (XLSX)
